# Determination of volatile organic compounds (VOCs) in indoor work environments by solid phase microextraction-gas chromatography-mass spectrometry

**DOI:** 10.1007/s11356-024-34715-7

**Published:** 2024-08-19

**Authors:** Wadir Mario Valentino Marchesiello, Giuseppina Spadaccino, Muhammad Usman, Donatella Nardiello, Maurizio Quinto

**Affiliations:** Department of Agriculture, Food, Natural Resources, and Engineering (DAFNE), University of Foggia, Via Napoli, 25, 71122 Foggia, Italy

**Keywords:** VOCs, Working environments, Indoor air quality, Industrial emissions, Solid phase microextraction (SPME), Gas chromatography-mass spectrometry (GC–MS)

## Abstract

Volatile organic compounds (VOCs) are continuously emitted into the atmosphere from natural and anthropogenic sources and rapidly spread from the atmosphere to different environments. A large group of VOCs has been included in the class of air pollutants; therefore, their determination and monitoring using reliable and sensitive analytical methods represents a key aspect of health risk assessment. In this work, an untargeted approach is proposed for the evaluation of the exposure to volatile organic compounds of workers in an engine manufacturing plant by GC–MS measurements, coupled with solid-phase microextraction (SPME). The analytical procedure was optimized in terms of SPME fiber, adsorption time, desorption time, and temperature gradient of the chromatographic run. For the microextraction of VOCs, the SPME fibers were exposed to the air in two different zones of the manufacturing factory, i.e., in the mixing painting chamber and the engine painting area. Moreover, the sampling was carried out with the painting system active and running (system on) and with the painting system switched off (system off). Overall, 212 compounds were identified, but only 17 were always present in both zones (mixing painting chamber and engine painting area), regardless of system conditions (on or off). Finally, a semi-quantitative evaluation was performed considering the peak area value of the potentially most toxic compounds by multivariate data analyses.

**Supplementary Information:**

The online version contains supplementary material available at 10.1007/s11356-024-34715-7.

## Introduction

The term VOCs (volatile organic compounds) covers a wide range of chemical classes (including aromatic and aliphatic amines, ethers, aldehydes, ketones, esters, alcohols, acids, and halogenated hydrocarbons) with high vapor pressure and low water solubility. The natural sources of VOCs are mainly the sea, vegetation, soil, volcanoes, and rainwater (Huang et al. 2018). Many VOCs are chemicals produced and used by humans in the preparation of pharmaceuticals, paints, and refrigerants. Numerous studies have reported that VOCs may have short- and long-term harmful effects on human health and cause respiratory, neurological, and circulation diseases (Kumar et al. 2018; Zhang et al. 2020). Furthermore, VOCs affect air quality and climate change as precursors of ground-level ozone and secondary organic aerosol (Guo et al. 2017).

Humans typically spend most of their time inside buildings, and this has prompted many field studies (Žitnik et al. 2010; Zhang and Zhu 2012). In closed environments, both residential and working, indoor air quality depends not only on building materials and furnishings (Schieweck and Salthammer 2009; Schieweck 2021), but also on human presence, the activity of occupants and their cleaning and personal care products (Tham 2016; González-Martín et al. 2021). Moreover, gases and/or particulates are also released from external sources, and all these aspects contribute to the overall VOC composition and air quality (Schalm et al. 2022).

VOC fingerprints are usually evaluated by gas chromatography coupled mass spectrometry and for the sample extraction, SPME (solid-phase microextraction) is one of the most used microextraction techniques for its ease of use and high extraction efficiency, in terms of the number and type of extracted compounds (Lord and Pawliszyn 2000; Nerín et al. 2009). SPME is a solvent-free technique that combines extraction, concentration, and clean-up into a single step, without requiring chemical-physical treatments; it is not invasive, and it does not change the environmental conditions of the sample, allowing the study of VOC production in real conditions (Ouyang and Pawliszyn 2006; Spadaccino et al. 2021).

In this work, an untargeted approach is described for the characterization of VOCs in indoor working environments by SPME–GC–MS. The exposure to volatile organic compounds of workers in different areas of an engine manufacturing plant was evaluated at different stages of the working cycle. Several SPME fibers of different polarity and composition were used to extract the greatest number of compounds, identified by mass spectrometry analysis coupled with database searching. Finally, multivariate analyses were performed to explore similarities and differences in the VOC composition.

## Materials and methods

### Solid-phase microextraction (SPME) experimental conditions

The microextraction process was carried out using an SPME device (Supelco, JVA Analytical Ltd., Ireland). Two fibers with different polarity were used: 85 µm carboxen/polydimethylsiloxane (CAR/PDMS), and 100 µm polydimethylsiloxane (PDMS), purchased from Supelco/Sigma-Aldrich (Bellefonte, PA, USA). The fibers were thermally conditioned following the manufacturer’s recommendations before their first use. Each SPME fiber was exposed to the air for 30 min, under controlled temperature (25 °C) and humidity (45%) conditions, with doors and windows closed, in two different areas of the manufacturing factory: in the mixing painting chamber (that is where the painting of the engines is carried out, area A) and the engine painting area (the working area adjacent to the engine painting room, zone B). The sampling was carried out with the factory active and running (hereinafter referred to as system on) and with the factory switched off (system off). The samples were analyzed in duplicate and blank runs were acquired before and after each GC–MS analysis.

### GC–MS analyses

GC–MS analyses were performed using an Agilent 7890 apparatus (Little Falls, DE, USA) coupled with an Agilent 5975 mass selective detector. The SPME device was directly inserted into the GC–MS injection port; the injections were made in splitless mode using an SPME injection sleeve (0.75 mm I.D) with a desorption temperature and time of 250 °C and 6 min, respectively. The chromatographic separations were carried out using the analytical J&W HP-5MS column (30 m × 0.25 mm I.D., 0.25 μm film thickness, Agilent Technologies, Santa Clara, CA, USA). The flow rate of the carrier gas (Helium, 99.999%) was 1.0 mL/min. The oven temperature was initially set at 45 °C for 5 min, then increased to 320 °C at a rate of 8 °C/min, and kept at 320 °C for 15 min before returning to the initial temperature, with a total cycle time of 54.36 min. The MS detector was operated in scan mode (mass range 45–300 m/z). MSD ChemStation (Agilent) was used for data acquisition and processing. Compound identifications were performed by comparing the experimental mass spectra to those contained in the National Institute of Standards and Technology database (NIST/EPA/NIHMass Spectral Library). The identity of the compound was confirmed only if a match quality score above 90 was achieved.

### Statistical analyses

In the absence of specific standards, since the compounds actually present in the analyzed samples are not known a priori, semi-quantitative measurements were carried out using peak area values of the identified compounds, which are directly related to their concentration. Therefore, for selected VOCs, among all the compounds identified in the indoor environmental samples, a semi-quantitative evaluation was performed considering the peak area value and, finally, multivariate statistical data analysis (principal component analysis) was performed by SIMCA® (Umetrics, Umea, Sweden).

## Results and discussion

### Optimization of SPME and chromatographic conditions

Several polymers with different thicknesses and polarity are commercially available and currently used as SPME coatings according to the analyte and sample characteristics. In this study, five different SPME coatings (i.e., polydimethylsiloxane, divinylbenzene, carboxen, polyacrylate, and polyethylene glycol) were tested and compared in terms of extraction efficiency, evaluated by the number of detected peaks (signal-to-noise ratio > 3) and the total peak area of the VOCs profile in air samples, used as a reference model, of an indoor work environment, within the same manufacturing plant, with the engine painting system turned off. Then, two fibers (monophasic and biphasic, based on polydimethylsiloxane and carboxen/polydimethylsiloxane, respectively) were chosen and used for all subsequent analyses to recover the greatest number of compounds. Therefore, for each air sample, two parallel 30 min-SPME extraction processes with the two different fibers were carried out and the total list of the identified compounds was obtained by combining the results of each analysis. The SPME procedure was also optimized in terms of adsorption and desorption time through a systematic investigation between 10–60 min (in 10-min steps) and 3–9 min (in 2-min steps), respectively. Then, by plotting the number of detected peaks and the total VOC peak area as a function of the parameter to be optimized (adsorption time and desorption time), optimal values of 30 min (adsorption time) and 6 min (desorption time) were determined and applied for all analyses.

**Fig. 1:**
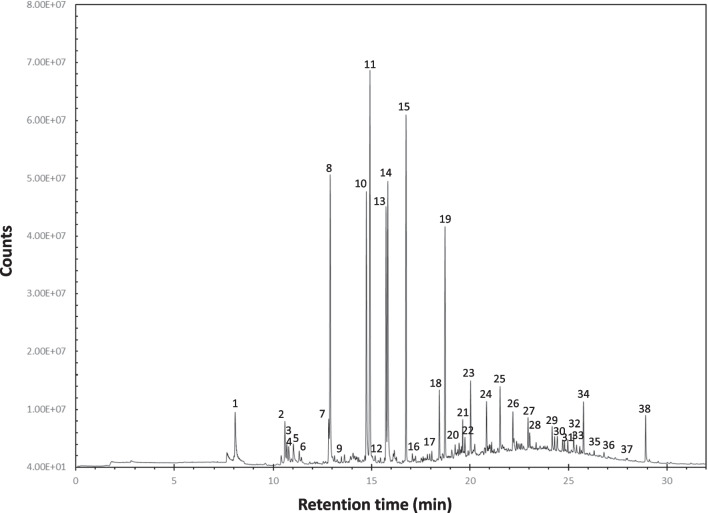
VOC chromatogram of zone A air sample -system ON obtained by SPME–GC–MS analysis in optimized operative conditions using the PDMS fiber. Most relevant compounds: (1) Ethanol, 2-butoxy-; (2) Benzene, 1,2,3-trimethyl-; (3) Decane; (4) 2-Propanol, 1-(2-methoxy-1-methylethoxy)-; (5) 1-Hexanol, 2-ethyl-; (6) Benzyl Alcohol; (7) Undecane; (8) Nonanal; (9) Dodecane; (10) Decanal; (11) Ethanol, 1-(2-butoxyethoxy)-; (12) Carbamic acid, ethyl-, methyl ester; (13) Tridecane; (14) Isooctyl mercaptoacetate; (15) 2,4-Decadienal, (E,E)-; (16) Naphthalene, 1,2,3,4-tetrahydro-1,4-dimethyl-; (17) Dodecane, 2,6,10-trimethyl-; (18) Tetradecane; (19) Ethanone, 1,1′-(1,4-phenylene)bis-; (20) 2,5-Cyclohexadiene-1,4-dione, 2,6-bis(1,1-dimethylethyl)-; (21) Pentadecane; (22) 4(1H)-Pteridinone, 2-amino-; (23) 2,5-Cyclohexadiene-1,4-dione, 2,6-bis(1,1-dimethylethyl)-; (24) 1,6-Dioxacyclododecane-7,12-dione; (25) Hexadecane; (26) Heptadecane, 7-methyl-; (27) 3,3′-Dimethylbiphenyl; (28) Hexadecane, 2,6,10-trimethyl-; (29) Benzenesulfonamide, N-butyl-; (30) Hexadecane; (31) Benzene, (1-pentyloctyl)-; (32) Benzene, (1-butylnonyl)-; (33) Benzene, (1-ethylundecyl)-; (34)Benzene, (1-methylundecyl)-; (35) n-Hexadecanoic acid; (36) Eicosane; (37) Heneicosane; (38) Phenol, 4,4′-(1-methylethylidene)bis-

The optimization of the temperature gradient was performed to achieve a good compromise between low chromatographic times and adequate peak resolution. Details of the employed analytical method are summarized in the experimental section, and, as an example, a typical VOC chromatographic profile is shown in Fig. 1.

### VOC profiles

The evaluation of indoor air quality was performed by a passive sampling process through SPME fiber exposure in different zones of the manufacturing factory. VOC measurements were conducted for three months during the summer, with a sampling frequency of 15 days; the total number of sampling times is 6, for each SPME fiber, both when the system is on and when the system is off. The complete list of the compounds identified in the inner and outer chamber, at system on and off, is shown in Table S1 (Online Resource – Supplementary Information). A total of 212 compounds were identified: 146 and 52 in Zone A at system ON and OFF, and 105 and 37 in Zone B at system ON and OFF, respectively.

As reported in Table 1, only 17 compounds were always present, both in Zone A and B, regardless of system conditions (ON or OFF). When the system was ON, 58 common compounds were observed both in Zone A and B (Table 2), while only 24 substances were concurrently found in both zones when the system was OFF (Table 3). Considering the Zone A, a compound comparison was performed between the system ON and OFF (Table 4). A total of 33 common compounds were found. Among them, higher levels of benzene and phenol-based molecules were observed when the system was ON. Similar evaluations were carried out about the data relating to the outer chamber (Table 5) at system on and off; fewer common compounds (i.e., 24) have been identified, at lower levels than those in the inner zone.

**Table 1:** List of common compounds found in the Zone A and Zone B at system ON and OFF

			Zone A		Zone B	
No	CAS No	Compound	System ON	System OFF	System ON	System OFF
65	4534–53-6	Benzene, (1-methyldodecyl)-	✓	✓	✓	✓
75	108–38-3	Benzene, 1,3-dimethyl-	✓	✓	✓	✓
86	100–51-6	Benzyl Alcohol	✓	✓	✓	✓
102	295–17-0	Cyclotetradecane	✓	✓	✓	✓
104	112–31-2	Decanal	✓	✓	✓	✓
109	112–40-3	Dodecane	✓	✓	✓	✓
117	112–95-8	Eicosane	✓	✓	✓	✓
119	112–34-5	Ethanol, 2-(2-butoxyethoxy)-	✓	✓	✓	✓
136	544–76-3	Hexadecane	✓	✓	✓	✓
151	57–10-3	n-Hexadecanoic acid	✓	✓	✓	✓
156	124–19-6	Nonanal	✓	✓	✓	✓
159	593–45-3	Octadecane	✓	✓	✓	✓
183	80–05-7	Phenol, 4,4′-(1-methylethylidene)bis-	✓	✓	✓	✓
190	106–42-3	p-Xylene	✓	✓	✓	✓
192	124–25-4	Tetradecanal	✓	✓	✓	✓
193	629–59-4	Tetradecane	✓	✓	✓	✓
204	1120–21-4	Undecane	✓	✓	✓	✓

**Table 2:** List of common compounds found in the Zone A and Zone B at system ON

			**System ON**	
**No**	**CAS No**	**Compound**	**Zone A**	**Zone B**
9	777–95-7	1,6-Dioxacyclododecane-7,12-dione	✓	✓
15	112–69-6	1-Hexadecanamine, N,N-dimethyl-	✓	✓
17	104–76-7	1-Hexanol, 2-ethyl-	✓	✓
18	3910–35-8	1H-Indene, 2,3-dihydro-1,1,3-trimethyl-3-phenyl-	✓	✓
24	719–22-2	2,5-Cyclohexadiene-1,4-dione, 2,6-bis(1,1-dimethylethyl)-	✓	✓
27	118–60-5	2-Ethylhexyl salicylate	✓	✓
53	99–93-4	Acetophenone, 4′-hydroxy-	✓	✓
59	55–21-0	Benzamide	✓	✓
62	4534–50-3	Benzene, (1-butylnonyl)-	✓	✓
64	4534–52-5	Benzene, (1-ethylundecyl)-	✓	✓
65	4534–53-6	Benzene, (1-methyldodecyl)-	✓	✓
66	4534–59-2	Benzene, (1-methyltridecyl)-	✓	✓
67	2719–61-1	Benzene, (1-methylundecyl)-	✓	✓
68	4534–49-0	Benzene, (1-pentyloctyl)-	✓	✓
69	4534–51-4	Benzene, (1-propyldecyl)-	✓	✓
72	526–73-8	Benzene, 1,2,3-trimethyl-	✓	✓
74	108–67-8	Benzene, 1,3,5-trimethyl-	✓	✓
75	108–38-3	Benzene, 1,3-dimethyl-	✓	✓
81	65–85-0	Benzenecarboxylic acid	✓	✓
82	3622–84-2	Benzenesulfonamide, N-butyl-	✓	✓
83	100–47-0	Benzonitrile	✓	✓
85	95–16-9	Benzothiazole	✓	✓
86	100–51-6	Benzyl Alcohol	✓	✓
91	14398–71-1	cis-Decalin, 2-syn-methyl-	✓	✓
102	295–17-0	Cyclotetradecane	✓	✓
104	112–31-2	Decanal	✓	✓
108	84–66-2	Diethyl Phthalate	✓	✓
109	112–40-3	Dodecane	✓	✓
111	3891–98-3	Dodecane, 2,6,10-trimethyl-	✓	✓
117	112–95-8	Eicosane	✓	✓
119	112–34-5	Ethanol, 2-(2-butoxyethoxy)-	✓	✓
120	111–76-2	Ethanol, 2-butoxy-	✓	✓
121	122–99-6	Ethanol, 2-phenoxy-	✓	✓
123	100–41-4	Ethylbenzene	✓	✓
127	629–94-7	Heneicosane	✓	✓
128	629–78-7	Heptadecane	✓	✓
136	544–76-3	Hexadecane	✓	✓
151	57–10-3	n-Hexadecanoic acid	✓	✓
156	124–19-6	Nonanal	✓	✓
157	112–05-0	Nonanoic acid	✓	✓
159	593–45-3	Octadecane	✓	✓
160	930–02-9	Octadecane, 1-(ethenyloxy)-	✓	✓
161	75163–97-2	Octadecane, 2,6-dimethyl-	✓	✓
167		Oxirane, heptadecyl-	✓	✓
171	95–47-6	o-Xylene	✓	✓
172	629–62-9	Pentadecane	✓	✓
175	6165–40-8	Pentadecane, 7-methyl-	✓	✓
178	108–95-2	Phenol	✓	✓
181	837–08-1	Phenol, 2,4′-isopropylidenedi-	✓	✓
183	80–05-7	Phenol, 4,4′-(1-methylethylidene)bis-	✓	✓
187	4286–23-1	p-Isopropenylphenol	✓	✓
190	106–42-3	p-Xylene	✓	✓
192	124–25-4	Tetradecanal	✓	✓
193	629–59-4	Tetradecane	✓	✓
194	544–63-8	Tetradecanoic acid	✓	✓
196	108–88-3	Toluene	✓	✓
200	629–50-5	Tridecane	✓	✓
203	121–44-8	Triethylamine	✓	✓
204	1120–21-4	Undecane	✓	✓

**Table 3:** List of common compounds found in the Zone A and Zone B at system OFF

			**System OFF**	
**No**	**CAS No**	**Compound**	**Zone A**	**Zone B**
65	4534–53-6	Benzene, (1-methyldodecyl)-	✓	✓
75	108–38-3	Benzene, 1,3-dimethyl-	✓	✓
86	100–51-6	Benzyl Alcohol	✓	✓
102	295–17-0	Cyclotetradecane	✓	✓
104	112–31-2	Decanal	✓	✓
109	112–40-3	Dodecane	✓	✓
117	112–95-8	Eicosane	✓	✓
119	112–34-5	Ethanol, 2-(2-butoxyethoxy)-	✓	✓
136	544–76-3	Hexadecane	✓	✓
151	57–10-3	n-Hexadecanoic acid	✓	✓
156	124–19-6	Nonanal	✓	✓
159	593–45-3	Octadecane	✓	✓
183	80–05-7	Phenol, 4,4′-(1-methylethylidene)bis-	✓	✓
190	106–42-3	p-Xylene	✓	✓
192	124–25-4	Tetradecanal	✓	✓
193	629–59-4	Tetradecane	✓	✓
204	1120–21-4	Undecane	✓	✓

**Table 4:** List of common compounds found in the Zone A at system ON and OFF

			**Zone A**	
**No**	**CAS No**	**Compound**	**System ON**	**System OFF**
9	777–95-7	1,6-Dioxacyclododecane-7,12-dione	✓	✓
15	112–69-6	1-Hexadecanamine, N,N-dimethyl-	✓	✓
44	3796–70-1	5,9-Undecadien-2-one, 6,10-dimethyl-, (E)-	✓	✓
65	4534–53-6	Benzene, (1-methyldodecyl)-	✓	✓
69	4534–51-4	Benzene, (1-propyldecyl)-	✓	✓
75	108–38-3	Benzene, 1,3-dimethyl-	✓	✓
82	3622–84-2	Benzenesulfonamide, N-butyl-	✓	✓
86	100–51-6	Benzyl Alcohol	✓	✓
102	295–17-0	Cyclotetradecane	✓	✓
104	112–31-2	Decanal	✓	✓
105	124–18-5	Decane	✓	✓
109	112–40-3	Dodecane	✓	✓
115	17312–57-1	Dodecane, 3-methyl-	✓	✓
117	112–95-8	Eicosane	✓	✓
119	112–34-5	Ethanol, 2-(2-butoxyethoxy)-	✓	✓
120	111–76-2	Ethanol, 2-butoxy-	✓	✓
127	629–94-7	Heneicosane	✓	✓
128	629–78-7	Heptadecane	✓	✓
136	544–76-3	Hexadecane	✓	✓
137	638–36-8	Hexadecane, 2,6,10,14-tetramethyl-	✓	✓
151	57–10-3	n-Hexadecanoic acid	✓	✓
156	124–19-6	Nonanal	✓	✓
157	112–05-0	Nonanoic acid	✓	✓
159	593–45-3	Octadecane	✓	✓
167		Oxirane, heptadecyl-	✓	✓
171	95–47-6	o-Xylene	✓	✓
181	837–08-1	Phenol, 2,4′-isopropylidenedi-	✓	✓
183	80–05-7	Phenol, 4,4′-(1-methylethylidene)bis-	✓	✓
190	106–42-3	p-Xylene	✓	✓
192	124–25-4	Tetradecanal	✓	✓
193	629–59-4	Tetradecane	✓	✓
200	629–50-5	Tridecane	✓	✓
204	1120–21-4	Undecane	✓	✓

**Table 5:** List of common compounds found in the Zone B at system ON and OFF

			**Zone B**	
**No**	**CAS No**	**Compound**	**System ON**	**System OFF**
59	55–21-0	Benzamide	✓	✓
64	4534–52-5	Benzene, (1-ethylundecyl)-	✓	✓
65	4534–53-6	Benzene, (1-methyldodecyl)-	✓	✓
75	108–38-3	Benzene, 1,3-dimethyl-	✓	✓
86	100–51-6	Benzyl Alcohol	✓	✓
93	296–56-0	Cycloeicosane	✓	✓
102	295–17-0	Cyclotetradecane	✓	✓
104	112–31-2	Decanal	✓	✓
109	112–40-3	Dodecane	✓	✓
110	112–52-7	Dodecane, 1-chloro-	✓	✓
117	112–95-8	Eicosane	✓	✓
119	112–34-5	Ethanol, 2-(2-butoxyethoxy)-	✓	✓
136	544–76-3	Hexadecane	✓	✓
151	57–10-3	n-Hexadecanoic acid	✓	✓
156	124–19-6	Nonanal	✓	✓
159	593–45-3	Octadecane	✓	✓
169	7320–37-8	Oxirane, tetradecyl-	✓	✓
172	629–62-9	Pentadecane	✓	✓
176	1002–84-2	Pentadecanoic acid	✓	✓
183	80–05-7	Phenol, 4,4′-(1-methylethylidene)bis-	✓	✓
190	106–42-3	p-Xylene	✓	✓
192	124–25-4	Tetradecanal	✓	✓
193	629–59-4	Tetradecane	✓	✓
204	1120–21-4	Undecane	✓	✓

**Fig. 2:**
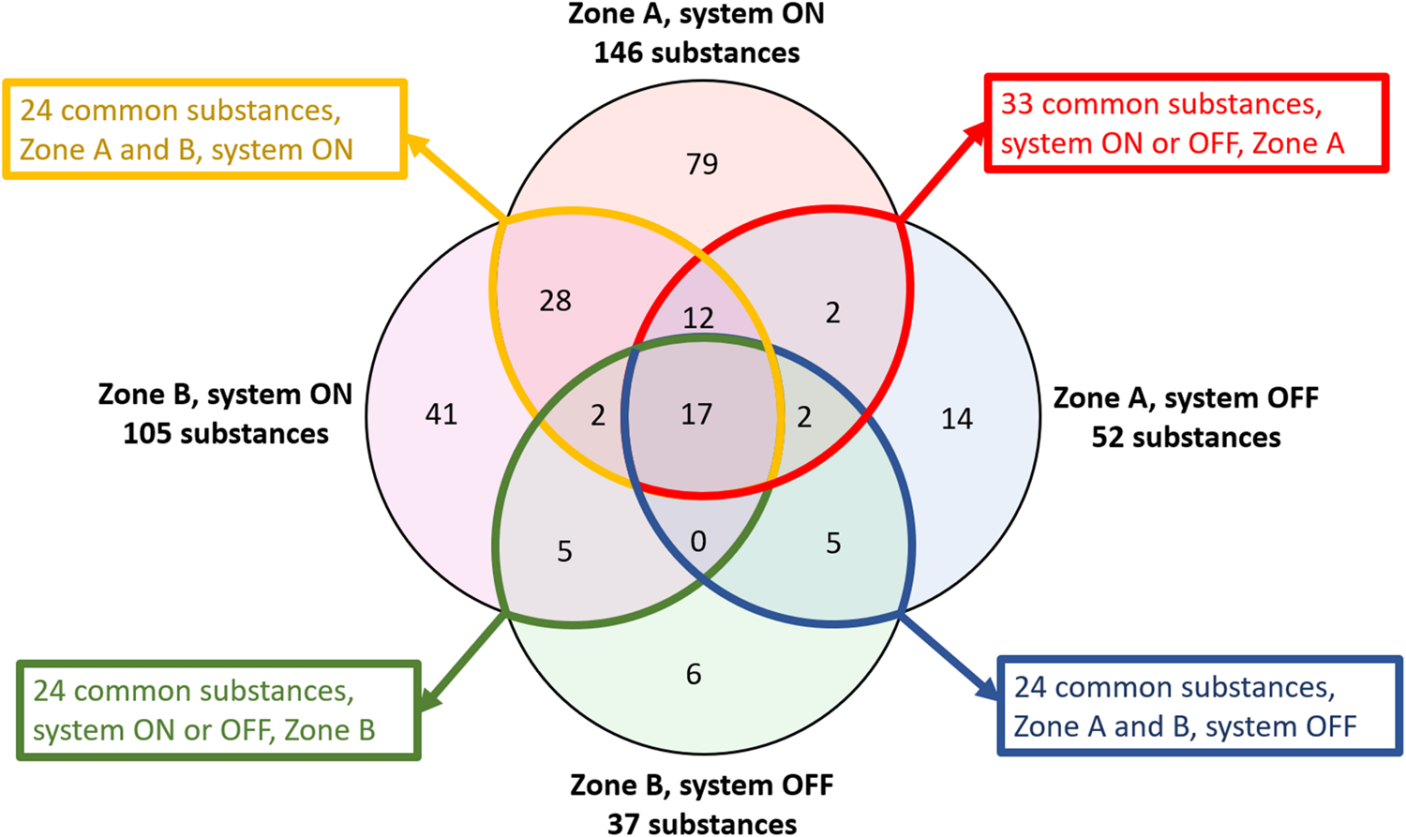
Venn diagram related to the number of identified compounds in different zone/system conditions, obtained by SPME–GC–MS

**Fig. 3:**
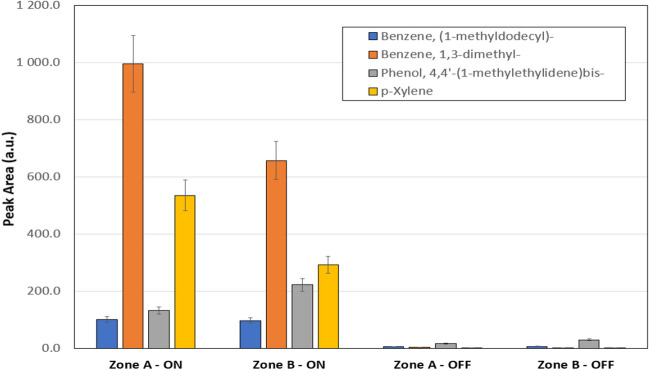
Peak area integration of the potentially toxic compounds identified in all cases (zone **A** and **B** and system ON and OFF)

A Venn diagram of the identified compounds, observed in specific conditions or common to the different situations, is shown in Fig. 2. Although mass concentrations of VOCs are important to get insight into toxicity and health risks, the aim of our work was not the quantitative determination of each VOC present in the environment, but the development of an optimized workflow to be used for air quality evaluation in health assessment studies. Therefore, semi-quantitative measurements were performed, considering that the peak area values of the identified compounds are directly related to their concentration levels. Among the 17 common compounds, identified in all cases (zone A and B and system ON and OFF), a semi-quantitative evaluation, reported in Fig. 3, was performed for benzene-based compounds (i.e., benzene, (1-methyldodecyl)-; benzene, 1,3-dimethyl-; phenol, 4,4′-(1-methylethylidene)bis-; and p-xylene), since benzene is classified as a carcinogen that increases the risk of cancer and other diseases, and is also a known cause of bone marrow failure. For these reasons, IARC (International Agency for Research on Cancer) has rated benzene as “known to be carcinogenic to humans”-Group 1, and human exposure to benzene is a global health problem. Additionally, long-term or repeated exposure to phenol and xylene may have harmful effects on the liver and kidneys and can also cause headaches, dizziness, confusion, loss of muscle coordination, and in high doses, death (Vyskocil et al. 2012).

**Fig. 4:**
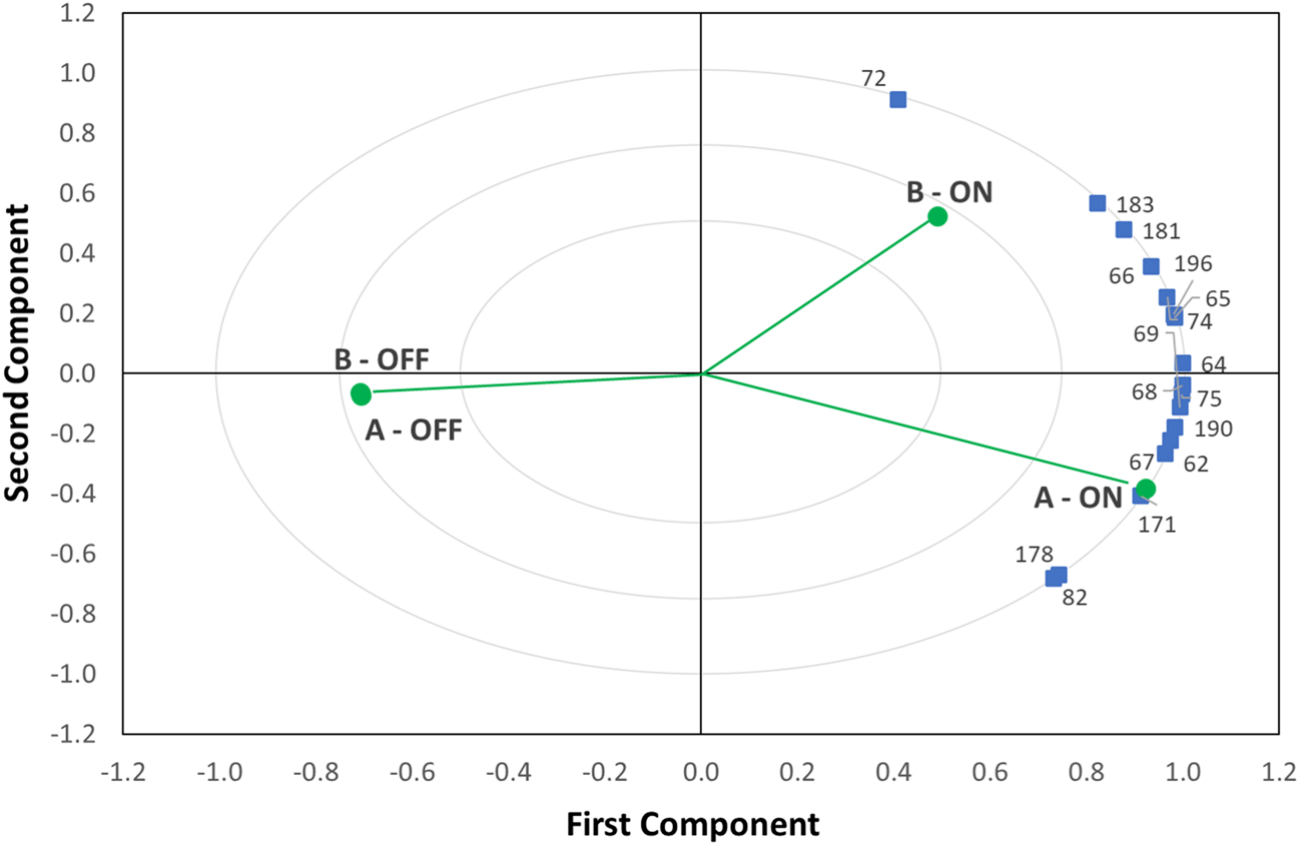
PCA biplot of air samples in different areas of the manufacturing factory (zones **A** and **B**) with the factory active (system ON) and switched off (system OFF)

Considering that the working conditions are different inside and outside the painting chamber when the system is on or off, multivariate analyses (i.e., Principal Component Analyses) were carried out to gain insight into these differences. The peak area values of a shortlist of 17 selected (presumably toxic) compounds such as benzene, xylene, and phenol derivatives, specifically: benzene, (1-butylnonyl)-; benzene, (1-ethylundecyl)-; benzene, (1-methyldodecyl)-; benzene, (1-methyltridecyl)-; benzene, (1-methylundecyl)-; benzene, (1-pentyloctyl)-; benzene, (1-propyldecyl)-; benzene, 1,2,3-trimethyl-; benzene, 1,3,5-trimethyl-; benzene, 1,3-dimethyl-; benzenesulfonamide, N-butyl-; o-xylene; phenol; phenol, 2,4′-isopropylidenedi-; phenol, 4,4′-(1-methylethylidene)bis-; p-xylene; toluene) were used for the statistical analysis. In the data matrix, empty gaps, corresponding to missing data for VOCs not found in some of the samples examined have been filled with a virtual peak area value equal to the LOD signal, estimated as the tenth part of the minimum peak area within the entire data set. After data pre-treatment (centering and normalization), the PCA model showed two principal components; the PC1 axis justifies 82% of the total variance, while PC2 covers a further 17%. Each variable positively affects the regression parameters of the PCA model (R^2^ = 0.99 and Q^2^ 0.99), and no outliers were found, as confirmed by the DModX plot (i.e., the distance to the model, measuring how well each observation fits the model). From the biplot of Fig. 4, it is possible to observe a dense distribution of all the compounds investigated in the region corresponding to the switched-on plant, along the outer edge. Indeed, as expected, the observations ON fall in the region occupied by a high number of variables, and, specifically, the Biplot region occupied by A and B OFF does not contain any compounds.

From the data in the correlation matrix, reported in Table S2 (Online Resource – Supplementary Information) Pearson coefficients higher than 0.90 were obtained for almost all compounds belonging to the class of benzene derivatives, except for the following three couples: benzene, (1-butylnonyl)-/benzene, 1,2,3-trimethyl-; benzene, (1-methylundecyl)-/benzene, 1,2,3-trimethyl-; and p-xylene/benzene, 1,2,3-trimethyl-. With the exception of two couples of compounds (phenol/benzene, 1,2,3-trimethyl-, and o-xylene/benzene, 1,2,3-trimethyl-) with negative Pearson coefficients, positive correlations between the two areas throughout the operating phases ON/OFF were observed for all other compounds. These outcomes suggest that, for these compounds (with strong positive correlations), high levels of one volatile compound mean high levels of another VOC. Therefore presumably, regardless of where they were found (A-ON, B-ON, A-OFF, B-OFF) these couples of compounds derived from the same emission source (volatilization of paint solvents, personal care products, building materials, etc.) and/or underwent similar processes, showing the same chemical fate and lifetime (Lu et al. 2022).

Although the total VOCs generally exhibit a seasonal pattern due to variations of sources and meteorological conditions, all our measurements were performed in summer, in the morning hours, when higher VOC concentrations appeared at industrial sites. Traffic emissions and solvent volatilization were the main sources of VOCs (Lu et al. 2022). Moreover, in summer, intensive photochemical reactions in the atmosphere can influence the VOC levels. Benzene and toluene belong to the class of the top 10 abundant species (Wu et al. 2020). Other important volatile compounds are ethylbenzene, p,m-xylene, and o-xylene, mainly coming from vehicular emissions and other combustion processes, specifically: industrial fuel combustion; vehicle exhausts including internal engine combustion and unburned emissions such as evaporation of vehicle fuels from motors and vehicle fuel tanks; liquefied petroleum gas leakage or natural gas leakage from vehicle tanks, cooking; biogenic emissions; use of solvent (Shi et al. 2022). In particular, benzene is closely correlated with wood combustion emissions (Xiong et al. 2020), while ethylbenzene, phenylethylene, and xylene isomers could result from the evaporation of solvents and paints.

The proposed experimental plan did not allow a quantitative analysis of each VOC found in the environment, but it allows the presence of these substances to be detected even at very low levels (ppb or ppt). Specifically, within the scope of this study, comparative evaluations between the levels of some selected volatile organic compounds in different working areas and stages were carried out. The results indicate, as expected, that when the manufacturing system is active, the relative amounts of organic compounds in the environment increase. Anyway, even in the absence of quantitative data, it can be assumed that the values obtained could be in line with the levels generally observed in urban areas, both residential and industrial sites, which depend on several environmental factors and human activities. Numerous studies have reported exposure to air pollutants and often refer to single cities, short time periods, and specific air pollutants (Fassò et al. 2023; Gilardi et al. 2023). In particular, the phenomena of air pollution from benzene-based molecules (benzene is a ubiquitous pollutant of indoor and outdoor air) have been extensively described in the recent literature, referring to various regions of Italy (Martellini et al. 2020; Toscano and Murena 2021; Cattaneo et al. 2021; Ielpo et al. 2021; Di Gilio et al. 2021; Cucciniello et al. 2022; Manco et al. 2022; Urbano et al. 2023). In addition to highly industrialized areas, high pollution levels are frequently reported in the most densely populated areas, in close correlation with geographic characteristics, climate, seasons, number of inhabitants, urban traffic, and time slots of the day (Battista et al. 2021; Badaloni et al. 2023; Ciacci et al. 2023). Therefore, in the complex air quality scenario, it is reasonable to assume that the levels of VOCs, found in indoor work environments, comply with occupational limits and air quality guidelines, taking into account the practices adopted by the company for continuous air exchange and all necessary measures for the health of workers, in full compliance with current legal provisions. Moreover, the effect of VOCs on indoor air quality and potential health consequences do not only depend on the concentration levels, but also on the exposure time which, generally, is limited to short periods of time for each worker, thanks to adequate work shifts.

## Conclusions

In this work, an untargeted approach has been successfully applied to evaluate the exposure to volatile organic compounds in indoor work environments. More than two hundred volatile compounds have been identified by SPME and GC–MS, coupled with database searching. A semi-quantitative comparison of VOC levels in different areas and work stages was carried out, followed by multivariate analyses to explore differences in the VOC composition.

As indoor air pollution has become a major health concern, the workplace monitoring by GC–MS fingerprinting, performed by the optimized workflow proposed in the present study, can be included within the consolidated approaches for the determination of air pollutants and the assessment of air quality in health assessment studies. Therefore, our study could be considered as the starting point for ad-hoc targeted quantitative determinations of specific VOCs, once identified as markers of air quality. Furthermore, although Italy, like every other European country, has adopted the European Legislative Decree 81/2008 to manage workplace safety, various commissions and research groups are working to update the legislation on indoor air quality; therefore, surveillance actions of internal environments and industrial emissions are continuously necessary.

## Supplementary Information

Below is the link to the electronic supplementary material.

Supplementary file1 (PDF 447 KB)

## Data Availability

The authors declare that the data supporting the findings of this study are available within the paper and its Supplementary Information files. Should any raw data files be needed in another format they are available from the corresponding author upon reasonable request.
