# Supplementary material for: Determination of volatile organic compounds (VOCs) in indoor work environments by solid phase microextraction-gas chromatography-mass spectrometry
Source: Environ Sci Pollut Res Int. 2024 Aug 19;31(40):52804–14. doi: 10.1007/s11356-024-34715-7 (PMC11379745; doi:10.1007/s11356-024-34715-7)
Supplement: Supplementary file 1 — Supplementary file1 (PDF 447 KB) [file 11356_2024_34715_MOESM1_ESM.pdf]

Environmental Science and Pollution Research

SUPPLEMENTARY INFORMATION

Determination of Volatile Organic Compounds (VOCs) in indoor working environments by Solid Phase Microextraction-Gas Chromatography-Mass Spectrometry

W.M. V. Marchesiello, G. Spadaccino, M. Usman, D. Nardiello*, M. Quinto

Department of Agriculture, Food, Natural resources and Engineering (DAFNE) - University of Foggia, Via Napoli, 25 - 71122 Foggia (Italy)

- **Table S1.** The total list of the compounds identified by SPME-GC-MS.
- **Table S2.** Correlation matrix obtained for selected compounds identified by SPME-GC-MS (see section 3.2 for details).

* Corresponding author phone: +39 0881 338173; email: donatella.nardiello@unifg.it

Table S1*. The total list of the compounds identified by SPME-GC-MS.*

			Zone A		Zone B	
No	CAS No	Compound	System ON	System OFF	System ON	System OFF
1	30689-78-2	(R)-(-)-(Z)-14-Methyl-8-hexadecen-1-ol			✓	
2	4254-15-3	(S)-(+)-1,2-Propanediol	✓			
3	80-56-8	alpha.-Pinene			✓	
4	37985-11-8	1-(4-Methylphenyl)-4-phenylbuta-1,3-diene	✓			
5	643-93-6	1,1'-Biphenyl, 3-methyl-	✓			
6	25395-31-7	1,2,3-Propanetriol, diacetate	✓			
7	4376-20-9	1,2-Benzenedicarboxylic acid, mono(2-ethylhexyl) ester	✓			✓
8	84-79-7	1,4-Naphthalenedione, 2-hydroxy-3-(3-methyl-2-butenyl)-		✓		
9	777-95-7	1,6-Dioxacyclododecane-7,12-dione	✓	✓	✓	
10	35289-31-7	11-Dodecenol	✓			
11	872-05-9	1-Decene	✓			
12	112-41-4	1-Dodecene	✓			
13	822-20-8	1-Heptadecanol, acetate			✓	
14	6765-39-5	1-Heptadecene			✓	
15	112-69-6	1-Hexadecanamine, N,N-dimethyl-	✓	✓	✓	
16	2917-26-2	1-Hexadecanethiol			✓	
17	104-76-7	1-Hexanol, 2-ethyl-	✓		✓	
18	3910-35-8	1H-Indene, 2,3-dihydro-1,1,3-trimethyl-3-phenyl-	✓		✓	
19	18435-45-5	1-Nonadecene	✓			
20	112-92-5	1-Octadecanol		✓		
21	112-88-9	1-Octadecene	✓			✓
22	7785-70-8	1R-.alpha.-Pinene			✓	
23	25152-84-5	2,4-Decadienal, (E,E)-	✓			
24	719-22-2	2,5-Cyclohexadiene-1,4-dione, 2,6-bis(1,1-dimethylethyl)-	✓		✓	
25	111-02-4	2,6,10,14,18,22-Tetracosahexaene, 2,6,10,15,19,23-hexamethyl-, (all-E)-	✓			
26	13150-81-7	2,6-Dimethyldecane	✓			
27	118-60-5	2-Ethylhexyl salicylate	✓		✓	
28	83834-59-7	2-Ethylhexyl trans-4-methoxycinnamate		✓		
29		2-Furanecarboxylic acid, 3,5-dimethylcyclohexyl ester	✓			
30	20324-32-7	2-Propanol, 1-(2-methoxy-1-methylethoxy)-	✓			
31	13429-07-7	2-Propanol, 1-(2-methoxypropoxy)-	✓			

No	CAS No	Compound	Zone A		Zone B	
			System ON	System OFF	System ON	System OFF
32	107-98-2	2-Propanol, 1-methoxy-			✓	
33	5466-77-3	2-Propenoic acid, 3-(4-methoxyphenyl)-, 2-ethylhexyl ester		✓		✓
34	26952-13-6	2-Tetradecene, (E)-	✓			
35	612-75-9	3,3'-Dimethylbiphenyl	✓			
36	14035-33-7	3,5-di-tert-Butyl-4-hydroxyacetophenone	✓			
37	1620-98-0	3,5-di-tert-Butyl-4-hydroxybenzaldehyde	✓			
38	498-60-2	3-Furaldehyde	✓			
39	726-18-1	4,4'-Diacetyldiphenylmethane			✓	
40	6004-38-2	4,7-Methano-1H-indene, octahydro-			✓	
41	4488-57-7	4,7-Methanoindene, 3a,4,5,6,7,7a-hexahydro-, endo-			✓	
42		4b,8-Dimethyl-2-isopropylphenanthrene, 4b,5,6,7,8,8a,9,10-octahydro-	✓			
43	6333-79-5	4-Chlorobenzenesulfonamide, N-methyl-	✓			
44	3796-70-1	5,9-Undecadien-2-one, 6,10-dimethyl-, (E)-	✓	✓		
45	88-29-9	7-Acetyl-6-ethyl-1,1,4,4-tetramethyltetralin		✓		
46	109680-01-5	7-Isopropyl-1,1,4a-trimethyl-1,2,3,4,4a,9,10,10a-octahydrophenanthrene	✓			
47	16269-05-9	8-Benzoyloctanoic acid	✓			
48	92618-89-8	Acetic acid, 1,7,7-trimethyl-bicyclo[2.2.1]hept-2-yl ester			✓	
49	123-86-4	Acetic acid, butyl ester			✓	
50	5348-82-3	Acetic acid, chloro-, octadecyl ester		✓		
51	140-11-4	Acetic acid, phenylmethyl ester	✓			
52	98-86-2	Acetophenone	✓			
53	99-93-4	Acetophenone, 4'-hydroxy-	✓		✓	
54	100-52-7	Benzaldehyde	✓			
55	15764-16-6	Benzaldehyde, 2,4-dimethyl-	✓			
56	100-83-4	Benzaldehyde, 3-hydroxy-	✓			
57	621-59-0	Benzaldehyde, 3-hydroxy-4-methoxy-			✓	
58	123-11-5	Benzaldehyde, 4-methoxy-	✓			
59	55-21-0	Benzamide	✓		✓	✓
60	71-43-2	Benzene	✓			
61	54986-44-6	Benzene, (1,3,3-trimethylnonyl)-		✓		
62	4534-50-3	Benzene, (1-butylnonyl)-	✓		✓	

			Zone A		Zone B	
No	CAS No	Compound	System ON	System OFF	System ON	System OFF
63	2400-00-2	Benzene, (1-ethyldecyl)-			✓	
64	4534-52-5	Benzene, (1-ethylundecyl)-	✓		✓	✓
65	4534-53-6	Benzene, (1-methyldodecyl)-	✓	✓	✓	✓
66	4534-59-2	Benzene, (1-methyltridecyl)-	✓		✓	
67	2719-61-1	Benzene, (1-methylundecyl)-	✓		✓	
68	4534-49-0	Benzene, (1-pentyloctyl)-	✓		✓	
69	4534-51-4	Benzene, (1-propyldecyl)-	✓	✓	✓	
70	700-60-7	Benzene, (trichloroethenyl)-	✓			
71		Benzene, 1,1'-(3,3-dimethyl-1-butenylidene)bis-	✓			
72	526-73-8	Benzene, 1,2,3-trimethyl-	✓		✓	
73	95-63-	Benzene, 1,2,4-trimethyl-	✓			
74	108-67-8	Benzene, 1,3,5-trimethyl-	✓		✓	
75	108-38-3	Benzene, 1,3-dimethyl-	✓	✓	✓	✓
76	7525-62-4	Benzene, 1-ethenyl-3-ethyl-	✓			
77	620-14-4	Benzene, 1-ethyl-3-methyl-			✓	
78	622-96-8	Benzene, 1-ethyl-4-methyl-			✓	
79	527-84-4	Benzene, 1-methyl-2-(1-methylethyl)-	✓			
80	103-65-1	Benzene, propyl-			✓	
81	65-85-0	Benzenecarboxylic acid	✓		✓	
82	3622-84-2	Benzenesulfonamide, N-butyl-	✓	✓	✓	
83	100-47-0	Benzonitrile	✓		✓	
84	119-61-9	Benzophenone	✓			
85	95-16-9	Benzothiazole	✓		✓	
86	100-51-6	Benzyl Alcohol	✓	✓	✓	✓
87		Bicyclo[10.8.0]eicosane, cis-				✓
88	5655-61-8	Bicyclo[2.2.1]heptan-2-ol, 1,7,7-trimethyl-, acetate, (1S-endo)-			✓	
89	76-49-3	Bornyl acetate			✓	
90	766-90-5	cis-.beta.-Methylstyrene	✓			
91	14398-71-1	cis-Decalin, 2-syn-methyl-	✓		✓	
92	294-62-2	Cyclododecane		✓	✓	
93	296-56-0	Cycloeicosane			✓	✓
94	295-65-8	Cyclohexadecane			✓	
95	4292-92-6	Cyclohexane, pentyl-	✓			

No	CAS No	Compound	Zone A		Zone B	
			System ON	System OFF	System ON	System OFF
96	2216-51-5	Cyclohexanol, 5-methyl-2-(1-methylethyl)-, [1R-(1.alpha.,2.beta.,5.alpha.)]-	✓			
97	301643-32-3	Cyclohexene, 4-(4-ethylcyclohexyl)-1-pentyl-	✓			
98	1222-05-5	Cyclopenta[g]-2-benzopyran, 1,3,4,6,7,8-hexahydro-4,6,6,7,8,8-hexamethyl-		✓	✓	
99	295-48-7	Cyclopentadecane	✓			
100		Cyclopentanecarboxamide, 3-ethenyl-2-(3-pentenylidene)-N-phenyl-, [1.alpha.,2Z(E),3.alpha.]-			✓	
101		Cyclopropane carboxamide, 2-cyclopropyl-2-methyl-N-(1-cyclopropylethyl)-	✓			
102	295-17-0	Cyclotetradecane	✓	✓	✓	✓
103		D,D-Dihomoandrostane, (5.alpha.)-	✓			
104	112-31-2	Decanal	✓	✓	✓	✓
105	124-18-5	Decane	✓	✓		✓
106	17312-53-7	Decane, 3,6-dimethyl-			✓	
107	17312-62-8	Decane, 5-propyl-	✓			
108	84-66-2	Diethyl Phthalate	✓		✓	
109	112-40-3	Dodecane	✓	✓	✓	✓
110	112-52-7	Dodecane, 1-chloro-			✓	✓
111	3891-98-3	Dodecane, 2,6,10-trimethyl-	✓		✓	
112	31295-56-4	Dodecane, 2,6,11-trimethyl-			✓	
113	1560-97-0	Dodecane, 2-methyl-		✓		
114	55045-08-4	Dodecane, 2-methyl-6-propyl-			✓	
115	17312-57-1	Dodecane, 3-methyl-	✓	✓		
116	143-07-7	Dodecanoic acid		✓		✓
117	112-95-8	Eicosane	✓	✓	✓	✓
118	54446-78-5	Ethanol, 1-(2-butoxyethoxy)-	✓			
119	112-34-5	Ethanol, 2-(2-butoxyethoxy)-	✓	✓	✓	✓
120	111-76-2	Ethanol, 2-butoxy-	✓	✓	✓	
121	122-99-6	Ethanol, 2-phenoxy-	✓		✓	
122	1009-61-6	Ethanone, 1,1'-(1,4-phenylene)bis-	✓			
123	100-41-4	Ethylbenzene	✓		✓	
124		Exo-tricyclo[5.2.1.0(2.6)]decane			✓	
125	98-01-1	Furfural	✓			

			Zone A		Zone B	
No	CAS No	Compound	System ON	System OFF	System ON	System OFF
126		Galaxolide 1		✓		
127	629-94-7	Heneicosane	✓	✓	✓	
128	629-78-7	Heptadecane	✓	✓	✓	
129	54833-48-6	Heptadecane, 2,6,10,15-tetramethyl-			✓	
130	20959-33-5	Heptadecane, 7-methyl-	✓			
131	13287-23-5	Heptadecane, 8-methyl-	✓			
132	7225-64-1	Heptadecane, 9-octyl-	✓			
133	506-12-7	Heptadecanoic acid				✓
134	5617-41-4	Heptylcyclohexane		✓		✓
135	629-80-1	Hexadecanal	✓			
136	544-76-3	Hexadecane	✓	✓	✓	✓
137	638-36-8	Hexadecane, 2,6,10,14-tetramethyl-	✓	✓		✓
138	55000-52-7	Hexadecane, 2,6,10-trimethyl-	✓			
139	1560-92-5	Hexadecane, 2-methyl-	✓			
140	112-39-0	Hexadecanoic acid, methyl ester	✓			
141	2416-20-8	Hexadecenoic acid, Z-11-	✓			
142	105-99-7	Hexanedioic acid, dibutyl ester		✓	✓	
143	149-57-5	Hexanoic acid, 2-ethyl-	✓			
144		Indolo[2,3-a]quinolizin-4(12H)-one, 1,2,3,6,7,12b-hexahydro-3,12b-dimethyl-			✓	
145	142-91-6	Isopropyl Palmitate	✓			
146	462-95-3	Methane, diethoxy-			✓	
147	124-10-7	Methyl tetradecanoate	✓			
148	4175-54-6	Naphthalene, 1,2,3,4-tetrahydro-1,4-dimethyl-	✓			
149	2958-76-1	Naphthalene, decahydro-2-methyl-			✓	
150	334-48-5	n-Decanoic acid	✓			
151	57-10-3	n-Hexadecanoic acid	✓	✓	✓	✓
152	629-92-5	Nonadecane	✓			
153	75163-99-4	Nonadecane, 2,3-dimethyl-	✓			
154	6418-45-7	Nonadecane, 3-methyl-	✓			
155	13287-24-6	Nonadecane, 9-methyl-	✓			
156	124-19-6	Nonanal	✓	✓	✓	✓
157	112-05-0	Nonanoic acid	✓	✓	✓	
158	638-66-4	Octadecanal			✓	

No	CAS No	Compound	Zone A		Zone B	
			System ON	System OFF	System ON	System OFF
159	593-45-3	Octadecane	✓	✓	✓	✓
160	930-02-9	Octadecane, 1-(ethenyloxy)-	✓		✓	
161	75163-97-2	Octadecane, 2,6-dimethyl-	✓		✓	
162	57-11-4	Octadecanoic acid			✓	
163	124-13-0	Octanal			✓	
164	2216-34-4	Octane, 4-methyl-	✓			
165	124-07-2	Octanoic Acid		✓		
166		Oxalic acid, dodecyl isobutyl ester			✓	
167		Oxirane, heptadecyl-	✓	✓	✓	
168	629-92-5	Oxirane, hexadecyl-		✓		
169	7320-37-8	Oxirane, tetradecyl-			✓	✓
170	18633-25-5	Oxirane, tridecyl-			✓	
171	95-47-6	o-Xylene	✓	✓	✓	
172	629-62-9	Pentadecane	✓		✓	✓
173	3892-00-0	Pentadecane, 2,6,10-trimethyl-	✓			
174	2801-87-8	Pentadecane, 4-methyl-	✓			
175	6165-40-8	Pentadecane, 7-methyl-	✓		✓	
176	1002-84-2	Pentadecanoic acid			✓	✓
177	1572-52-7	Pentanedinitrile, 2-methylene-	✓			
178	108-95-2	Phenol	✓		✓	
179		Phenol, 2-(1,1-dimethylethyl)-4-(1-methyl-1-phenylethyl)-			✓	
180	96-76-4	Phenol, 2,4-bis(1,1-dimethylethyl)-			✓	
181	837-08-1	Phenol, 2,4'-isopropylidenedi-	✓	✓	✓	
182	5875-45-6	Phenol, 2,5-bis(1,1-dimethylethyl)-			✓	
183	80-05-7	Phenol, 4,4'-(1-methylethylidene)bis-	✓	✓	✓	✓
184	98-54-4	Phenol, p-tert-butyl-		✓		
185	85-44-9	Phthalic anhydride	✓			
186		Piperidine, 1-(5-trifluoromethyl-2-pyridyl)-4-(1H-pyrrol-1-yl)-			✓	
187	4286-23-1	p-Isopropenylphenol	✓		✓	
188	79-09-4	Propanoic acid	✓			
189	2078-54-8	Propofol	✓			
190	106-42-3	p-Xylene	✓	✓	✓	✓
191	100-42-5	Styrene	✓			

			Zone A		Zone B	
No	CAS No	Compound	System ON	System OFF	System ON	System OFF
192	124-25-4	Tetradecanal	✓	✓	✓	✓
193	629-59-4	Tetradecane	✓	✓	✓	✓
194	544-63-8	Tetradecanoic acid	✓		✓	
195	852228-22-9	Tetrapentacontane, 1,54-dibromo-	✓			
196	108-88-3	Toluene	✓		✓	
197		trans, cis-3-Ethylbicyclo[4.4.0]decane	✓			
198		trans-Decalin, 2-methyl-	✓			
199	103495-51-8	Tricyclo[3.1.0.0(2,4)]hex-3-ene-3-carbonitrile	✓			
200	629-50-5	Tridecane	✓	✓	✓	
201	1560-96-9	Tridecane, 2-methyl-	✓			
202	55045-11-9	Tridecane, 5-propyl-	✓			
203	121-44-8	Triethylamine	✓		✓	
204	1120-21-4	Undecane	✓	✓	✓	✓
205	17301-23-4	Undecane, 2,6-dimethyl-		✓		✓
206	17312-82-2	Undecane, 4,6-dimethyl-				✓
207	112-37-8	Undecanoic acid	✓			
208	121-33-5	Vanillin	✓			
209	19814-75-6	Xanthene, 9,9-dimethyl-			✓	
210		Z-11(13,13-Dimethyl)tetradecen-1-ol acetate	✓			
211	76402-60-3	Z-5-Nonadecene		✓		✓
212	2416-19-5	Z-7-Hexadecenoic acid				✓
		TOTAL	146	52	105	37

Table S2. Correlation matrix obtained for selected compounds identified by SPME-GC-MS (see section 3.2 for details).

**ID compound**	62	64	65	66	67	68	69	72	74	75	82	171	178	181	183	190	196
62	1	0.966457	0.915875	0.830693	0.999003	0.982749	0.993249	0.193171	0.88567	0.987681	0.86574	0.981132	0.873606	0.747073	0.67419	0.998949	0.911876
64		1	0.988113	0.945435	0.95406	0.997158	0.98834	0.438016	0.974896	0.994612	0.708285	0.898673	0.719652	0.891105	0.841254	0.977167	0.986429
65			1	0.984277	0.897045	0.97431	0.954787	0.570751	0.997535	0.967392	0.591989	0.82098	0.604784	0.950136	0.913441	0.933304	0.999921
66				1	0.805014	0.919326	0.887918	0.706711	0.994218	0.907575	0.440638	0.707383	0.454774	0.989873	0.969929	0.855338	0.986008
67					1	0.973514	0.987221	0.149182	0.864062	0.979712	0.887209	0.988784	0.894457	0.71672	0.640655	0.995907	0.892645
68						1	0.996987	0.371299	0.956264	0.999582	0.758281	0.92845	0.768536	0.85686	0.798721	0.990193	0.972058
69							1	0.302602	0.932094	0.998703	0.803817	0.952725	0.812787	0.818061	0.75116	0.997399	0.952045
72								1	0.626656	0.344322	-0.32363	-0.00017	-0.30871	0.795128	0.852601	0.237941	0.578883
74									1	0.947414	0.534469	0.77919	0.547776	0.969644	0.938954	0.906022	0.998207
75										1	0.776808	0.938794	0.786697	0.841711	0.781023	0.993816	0.964871
82											1	0.946149	0.999773	0.315551	0.214505	0.84191	0.584097
171												1	0.951206	0.604796	0.519047	0.971239	0.815313
178													1	0.329859	0.2307	0.850382	0.596874
181														1	0.989465	0.776726	0.953503
183															1	0.70718	0.916976
190																1	0.929732
196																	1
